# Adverse events in neurosurgery: a comprehensive single-center analysis of a prospectively compiled database

**DOI:** 10.1007/s00701-022-05462-w

**Published:** 2023-01-10

**Authors:** Philip Dao Trong, Arturo Olivares, Ahmed El Damaty, Andreas Unterberg

**Affiliations:** Department of Neurosurgery, University Hospital Heidelberg, Im Neuenheimer Feld 400, 69120 Heidelberg, Germany

**Keywords:** Adverse events, Morbidity and mortality, Neurosurgery, Post-operative complications, Quality management

## Abstract

**Purpose:**

To prospectively identify and quantify neurosurgical adverse events (AEs) in a tertiary care hospital.

**Methods:**

From January 2021 to December 2021, all patients treated in our department received a peer-reviewed AE-evaluation form at discharge. An AE was defined as any event after surgery that resulted in an undesirable clinical outcome, which is not caused by the underlying disease, that prolonged patient stay, resulted in readmission, caused a new neurological deficit, required revision surgery or life-saving intervention, or contributed to death. We considered AEs occurring within 30 days after discharge. AEs were categorized in wound event, cerebrospinal fluid (CSF) event, CSF shunt malfunction, post-operative infection, malpositioning of implanted material, new neurological deficit, rebleeding, and surgical goal not achieved and non-neurosurgical AEs.

**Results:**

2874 patients were included. Most procedures were cranial (45.1%), followed by spinal (33.9%), subdural (7.7%), CSF (7.0%), neuromodulation (4.0%), and other (2.3%). In total, there were 621 AEs shared by 532 patients (18.5%). 80 (2.8%) patients had multiple AEs. Most AEs were non-neurosurgical (222; 8.1%). There were 172 (6%) revision surgeries. Patients receiving cranial interventions had the most AEs (19.1%) although revision surgery was only necessary in 3.1% of patients. Subdural interventions had the highest revision rate (12.6%). The majority of fatalities was admitted as an emergency (81/91 patients, 89%). Ten elective patients had lethal complications, six of them related to surgery (0.2%).

**Conclusion:**

This study presents the one-year results of a prospectively compiled AE database. Neurosurgical AEs arose in one in five patients. Although the need for revision surgery was low, the rate of AEs highlights the importance of a systematic AE database to deliver continued high-quality in a high-volume center.

## Introduction

Morbidity and mortality conferences (MMC) evolved in the last decades as a tool by which surgeons could analyze complications to better understand potential causes of individual or system failures and to implement modifications that prevent their repeated occurrences. The information acquired through these conferences can significantly reduce “avoidable” adverse events in both residents and experienced surgeons [12]. Especially neurosurgical patients are prone to neurological morbidity [10, 16, 19]. These have major implications for patients and their families and represent a major burden to health care systems as some deficits may not resolve [17]. To fully elucidate the risks and benefits of neurosurgical procedures, it is mandatory to specify the rate of adverse events at an institutional level [20]. Not only is this data necessary to deliver a transparent informed consent to the patient but it is also an indispensable marker for modern day hospital management to continuously monitor adverse events and identify potential risk factors [5, 29]. Especially high-volume centers are facing several challenges when generating these data. Larger studies are mostly retrospective in character and often utilize administrative hospital data such as readmission or reoperation events and are therefore prone to underreporting [2, 19, 21, 23, 25]. Adverse events should be reported in a prospective and standardized manner across multiple neurosurgeons but only few studies have addressed this appropriately [1, 4, 15, 16, 18, 22, 27]. Other studies focus on a specific pathology or treatment only [7, 30–34]. Also, the implementation of a peer-review evaluation process is essential to further reduce subjective bias. Most importantly, to improve patient care, regular morbidity and mortality conferences are to be held for training purposes [8]. To add further body of evidence on adverse events in neurosurgery, this study presents a comprehensive analysis of a prospectively compiled database of a large neurosurgical center.

## Material and methods

### Study design

**Fig. 1:**
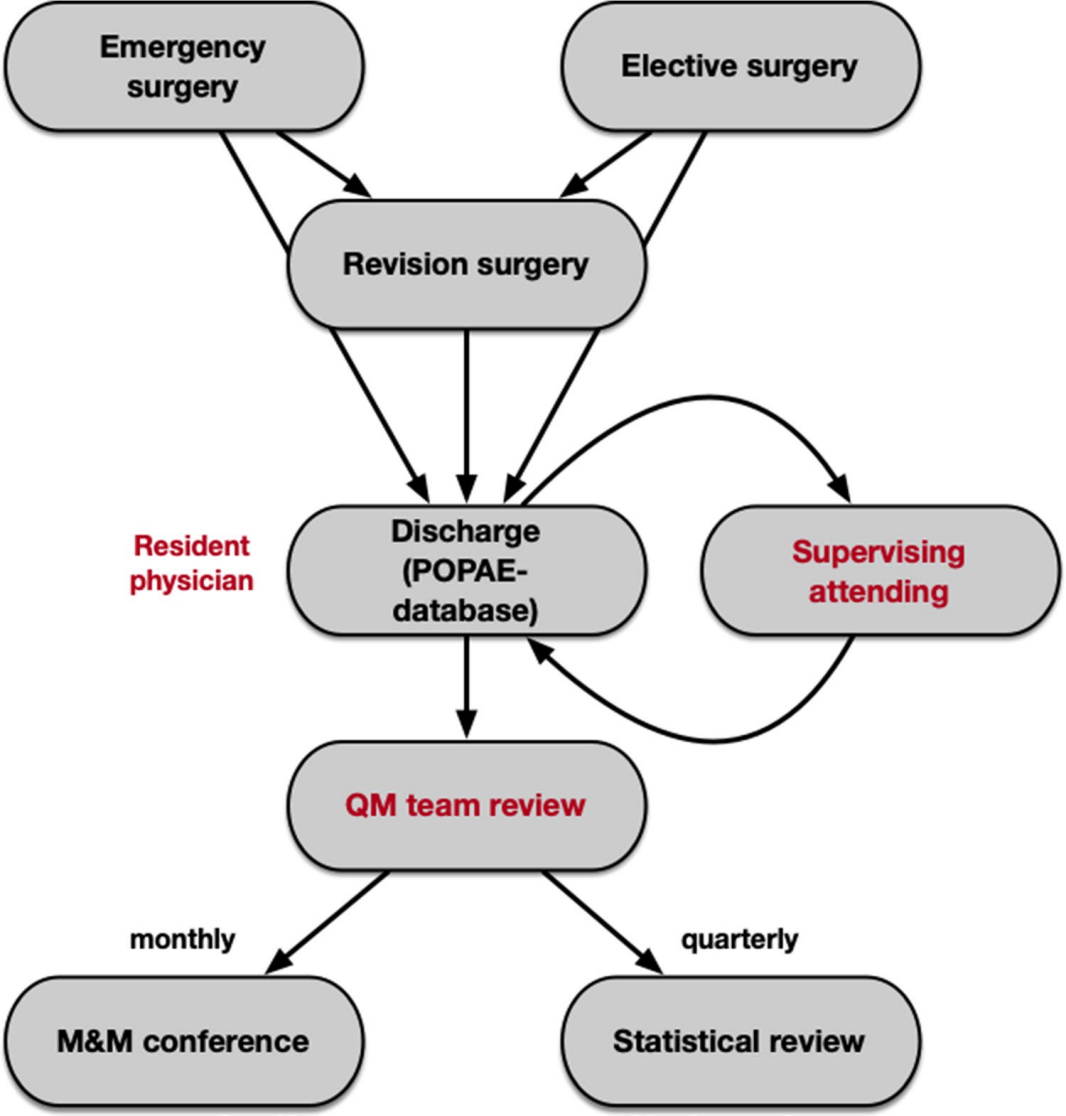
Prospective data acquisition algorithm. All patients treated in our neurosurgical department receive a post-operative adverse event evaluation form (POPAE) upon discharge filled out and reviewed by the resident physician and supervising attending. Regular morbidity and mortality conferences as well as statistical analyses are performed by the QM team

This study analyzes a prospectively compiled database at a single neurosurgical tertiary care hospital. 15 board certified and 18 resident neurosurgeons continuously contribute to the database. Every patient receives a post-operative adverse event (POPAE) form when discharged, filled out by the responsible neurosurgical physician of the ward. The form is then handed to the supervising senior attending for review. Only upon approval is the data fed into the POPAE database. If a patient gets readmitted within 30 days after initial surgery, an automatic warning will be passed on to the treating team. On a regular basis, relevant cases are being presented to the staff and thoroughly discussed. Statistics are being reviewed quarterly for anomalies and also presented to the staff (Fig. 1). For this study, the amount of revision surgeries within 30 days was compared between our POPAE database and the hospital administration database for cross referencing and deemed consistent. Approval from the ethics committee of the Medical Faculty of the University of Heidelberg was obtained (reference S-425/2022) and is in line with the principles of the Declaration of Helsinki.

### Definitions

Adverse events were categorized as defined in Table 1. Elective surgery was defined by any intervention that was scheduled at least one day beforehand. Non-elective surgery comprised emergency procedures and revision surgery. Cranial surgeries include all pathologies requiring craniotomy, stereotactic biopsies, endoscopic, and transsphenoidal procedures. Spinal surgeries include all degenerative, traumatic, oncologic, and pediatric malformation cases. Cerebrospinal fluid (CSF) procedures include all kinds of temporary or permanent CSF diversion procedures. Subdural procedures include all interventions needing only access to the subdural space. Neuromodulation includes all cases where thermal, chemical, or electrical energy is applied to neural structures. Other surgeries include muscle biopsies, peripheral nerve surgery, and cutaneous lipomas.

**Table 1:** Categorization of adverse events

Adverse event category	Defintion
Wound event	Superficial or deep wound healing issues with or without infection
Post-operative infections	Meningitis, abscess, or empyema formation
CSF fistula	Internal or external CSF fistula and rhinoliquorrhea
Implant malfunction/CSF shunt dysfunction	Valvular dysfunction, mechanical obstruction, and catheter occlusion
Malpositioning of implanted material	Ventricular or abdominal CSF catheter malpositioning, pedicle screw or rod misplacement, and intervertebral cage misplacement
New neurological deficit	New neurological deficit not present preoperatively and aggravation of neurological deficits
Rebleeding	Any rebleeding into resection cavity, subdural space, and soft tissue resulting in a new neurological deficit or revision surgery
Surgical goal not achieved	Preoperative goal not achieved and incomplete surgery

A neurosurgical adverse event (AE) was defined as any event that occurred within 30 days of initial surgery, that.

- Resulted in an undesirable clinical outcome which is not caused by the underlying disease
- Required a prolonged hospital stay as deemed by the supervising team
- Required readmission
- Required revision surgery or life-saving intervention
- Resulted in a new reversible or irreversible neurological deficit at time of discharge
- Or contributed to death

Non-neurosurgical AEs were analyzed for all patients who were admitted on the normal ward for an elective intervention. They were defined as any event that was not directly related to the planned neurosurgical procedure and occurred after admission. We excluded minor medical adverse events which did not have a significant impact on the clinical course such as urinary tract infections or electrolyte imbalances. Non-neurosurgical AEs that arose in patients who were admitted as an emergency were excluded because of the nature of the underlying pathology having per se a high rate of medical AEs (e.g., ventilator associated pneumonia and opioid induced bowel disorder).

Therefore, only severe medical AEs for patients who were admitted to the normal ward for an elective intervention which

- Prolonged the hospital stay on our normal ward
- Needed secondary transfer to our intermediate or intensive care unit
- Or resulted in death

were included in this study.

## Results

### Patient characteristics

**Fig. 2:**
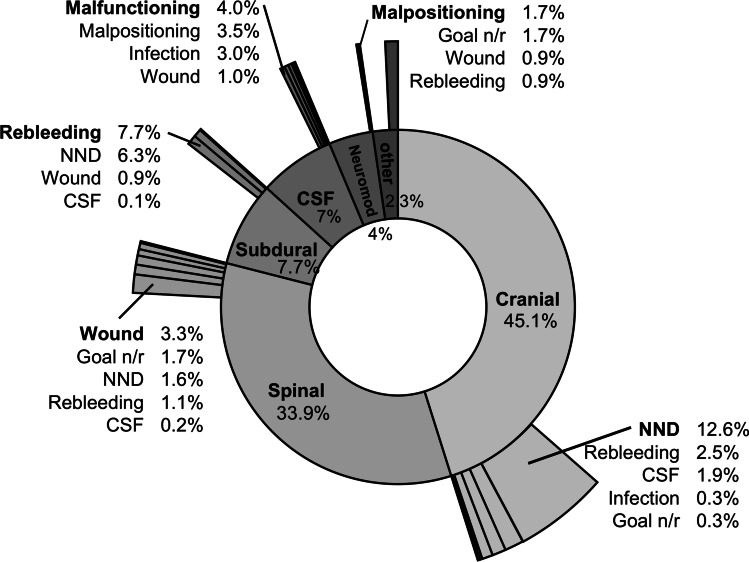
Summary of neurosurgical procedures and its adverse events (non-neurosurgical AEs excluded). CSF: cerebrospinal fluid event; Goal n/r: surgical goal not achieved; Infection: post-operative infection; Malfunctioning: CSF shunt malfunction; Malpositioning: malpositioning of implanted material; Neuromod: neuromodulation; NND: new neurological deficit

2874 patients were operated between January 2021 and December 2021. 2192 (76.3%) patients were treated electively, and 547 (19%) patients received emergency procedures (Table 2). Most patients received cranial surgery (45%) followed by spinal (34%), subdural (8.1%), and CSF (7.3%) interventions (Fig. 2). Neuromodulatory interventions accounted for 4.2% of all cases. Mean age was highest in the subdural patient group (74 years) and lowest in the CSF patient cohort (36 years).

**Table 2:** Patient characteristics

	*n* =	% of total	Mean age
Total interventions	2874		57
Elective	2327	81.0	56
Non-elective	547	19.0	60
Cranial	1297	45.1	54
Spinal	973	33.9	60
Subdural	222	7.7	74
CSF	200	7.0	36
Neuromodulation	116	4.0	56
Other	66	2.3	53

### Adverse events

In total, 532 (18.5%) patients shared 621 AEs (Fig. 2, Table 3). Of these, 80 (2.8%) patients had more than one AE. 222 (8.1%) AEs were not directly related to the neurosurgical procedure. There were 172 (6.0%) unplanned returns to the OR within the first 30 days after initial surgery (Table 3). Patients receiving cranial interventions had the highest number of AEs (19.1%) although revision surgery was only necessary in 3.1% of patients. CSF procedures had an AE rate of 10% and a revision rate of 7.5%. Subdural interventions (189 chronic subdural hematoma (SDH), 31 acute SDH, 1 subdural empyema, and 1 subdural hygroma) had an AE rate of 15.3% with the highest revision rate of 13.1%. Spinal interventions had a cumulative AE rate of 9.2% with a revision rate of 1.6%.

**Table 3:** Summary of adverse events

	*n* =	%	Revision surgery	%
Wound event				
Cranial	19	1.5	17	1.3
Spinal	32	3.3	15	1.5
Neuromodulation	1	0.9	1	0.9
Subdural	2	0.9	1	0.5
CSF	2	1.0	1	0.5
Other	2	3.0	1	1.5
Total	58	2.1	36	1.3
Postoperative infection				
CSF	6	3.0	5	2.5
Cranial	4	0.3	4	0.3
Spinal	2	0.2	2	0.2
Total	12	0.4	11	0.4
CSF event				
Cranial	24	1.9	18	1.4
Spinal	11	1.1	11	1.1
Subdural	1	0.1	1	0.5
Total	36	1.3	30	1.1
CSF shunt malfunction				
Total	8	4.0	8	4.0
Malpositioning of implanted material				
Neuromodulation	2	1.7	1	0.9
CSF	7	3.5	7	3.5
Spinal	1	0.5	1	0.1
Total	10	0.4	10	0.4
New neurological deficit				
Cranial	164	12.6	24	1.9
Spinal	16	1.6	3	0.3
CSF	2	1.0	1	0.5
Subdural	14	6.3	12	5.4
Total	196	7.2	40	1.5
Rebleeding				
Cranial	33	2.5	25	1.9
Spinal	11	1.1	8	0.8
Neuromodulation	1	0.9	1	0.9
subdural	17	7.7	15	6.8
CSF	3	1.5	1	0.5
Total	65	2.4	49	1.8
Surgical goal not achieved				
Cranial	4	0.3	2	0.2
Spinal	17	1.7	7	0.7
Neuromodulation	2	1.7	2	1.7
Total	23	0.8	11	0.4
Others				
Total	22	0.8	2	0.1
Non-neurosurgical AEs in elective surgery				
Prolonged hospital stay on normal ward	57	2.6		
Secondary transfer to IMC or ICU	33	1.5		
Lethal	10	0.5		
Total	100	4.6		

*CSF*, cerebrospinal fluid; *IMC*, intermediate care unit; *ICU*, intensive care unit

#### Wound related AEs (wound AE, post-operative infection, and CSF event)

Wound events were highest among spinal procedures and twice as often as in cranial interventions (3.3% vs. 1.5%). The category “postoperative infection,” which comprised abscess and empyema formation as well as CSF involvement, was treated separately because of the severity of the infection and the invasiveness of the subsequent treatment. Here, post-operative infections were most common in CSF diversion procedures (3%), requiring revision surgery in almost all cases. CSF events were most common in cranial procedures (1.9%).

#### New neurological deficit

New neurological deficits were most common following cranial procedures (12.6%), although revision surgery was needed only in 1.9% (24) of patients. These consisted mainly of hematoma evacuations, secondary decompressive surgery due to infarctions and secondary hydrocephalus treatment. 16 patients receiving spinal procedures had a post-operative deterioration of their neurological status. 13 patients showed new or aggravated radicular deficits which were treated conservatively. 3 patients received revision surgery where an extension of decompression was necessary.

#### Rebleeding

Subdural procedures were most likely to rebleed (17/222; 7.7%) and had to undergo repeated surgery in 15 cases (6.8%). The remaining 2 cases had bleeding locations distant from the site of surgery, where evacuation was not deemed necessary. 33 (2.5%) cranial and 11 (1.1%) spinal interventions rebled needing reintervention in 25 (1.9%) and 8 (0.8%) cases, respectively.

#### Surgical goal not achieved

This category summarizes interventions where the pre-operative objective was not achieved. In the cranial subsection, 3 patients had to undergo repeated surgery because of remaining tumor burden. In the spinal category, 17 patients did not benefit as expected from decompressive surgery at time of discharge and 7 patients received further or repeated decompressive surgery within the first 30 days after initial surgery.

#### Non-neurosurgical adverse events

Of the 2192 patients being electively operated in our department, 70 (3.2%) patients had a non-neurosurgical AE as defined above. 33 (1.5%) patients were transferred to our intermediate care unit (IMC) or intensive care unit (ICU) for mild to severe AEs. The most common non-neurosurgical AEs were pulmonary events (9), cardiac events (8), thromboembolic events (7), and septic events (6).

### Mortality

In our study cohort, 91 patients died. Most of the patients (81/91; 89%) were admitted as an emergency. Of these, 52 patients died of direct consequences of the underlying neurosurgical pathology (e.g., severe traumatic brain injury, intracerebral hemorrhage, subarachnoid hemorrhage, and uncontrollable intracranial pressure). The remaining emergency patients (29) died of medical reasons (e.g., septic shock, pulmonary thromboembolism, and cardiac insufficiency). 10 patients who received elective surgery had lethal complications. 3 patients died because of medical reasons (pulmonary thromboembolism, aspiration pneumonia, and GI bleeding). 1 patient died of the underlying disease (meningeosis carcinomatosa). Only 6 patients had lethal complications directly related to surgery (postoperative rebleeding and cerebral ischemia).

## Discussion

Identifying and evaluating neurosurgical adverse events in high-volume centers remain challenging. This study provides a one-year analysis of a prospectively compiled database of adverse events which occurred within 30 days after discharge of 2874 patients treated in our institution. In our study cohort, one in five patients is at risk of developing an adverse event.

Large studies analyzing adverse events in neurosurgical practice are often retrospective in character and often use administrative data proxies such as the 30-day readmission or reoperation rate [2, 19, 23, 24]. These parameters are solid surrogate markers for major adverse events needing further interventions or surveillance and are used to calculate reimbursement rates [35]. As health care costs in Germany are steadily rising and have almost doubled in the last 20 years from 2724 EUR per capita and year in 2001 to 5298 EUR in 2021 (Federal Statistical Office of Germany), the government’s responsibility to assess avertible health care costs to improve cost effectiveness has become more important than ever to alleviate societies health care burden. As cost effectiveness is defined as the patient’s outcome relative to the cost of care, adverse events represent an important variable to be accounted for. Nevertheless, these administrative parameters do not reflect healthcare outcomes of individual patients in all its dimensions [21]. AEs which do not fall into these categories, e.g., AEs which happen during the initial stay without the need for reoperation or readmission, are not accurately represented. To address this issue adequately, prospective institutional databases are needed.

First, it is indispensable to categorize the wide spectrum of neurosurgical procedures since adverse events have a different impact on different procedures. For example, infections in spinal surgery often result in antibiotic treatment and local revision surgery whereas infections after CSF procedures need utmost care and attention as it may lead to a life-threatening ventriculitis or meningitis. Large retrospective studies often only discriminate between cranial and spinal interventions [16, 19, 27]. To account for this shortcoming, we delineated our procedures in more detail. Procedures that needed access only to the subdural space (acute or chronic subdural hematomas and hygromas) and CSF diversion procedures were separated from cranial cases, because they entail a special patient population with a specific AE profile. As expected, the mean age was significantly higher in the subdural patient cohort compared to all other categories (mean age: 74 years). Inversely, patients needing CSF diversion procedures were significantly younger (mean age 36 years). Subdural procedures had the greatest number of cumulative AEs (15.3%) with a reoperation rate of 7.5%. This is in line with reports in the literature (4.2–22%), although most of the studies focused on the treatment of chronic subdural hematomas only [7, 9, 11]. CSF procedures had the second highest reoperation rate (7.5%). This is in line with studies analyzing AEs in CSF procedures [6, 30]. Valvular or mechanical dysfunction was the most common AEs ranging from 8 to 64%. The wide range is due to the different patient cohorts studied and varying follow-up times (pediatric vs. mixed/adults; follow-up 6 months to several years). In our series, shunt malfunction, infection, and hemorrhage in CSF procedures were lower than reported in the literature. This circumstance may be due to the shorter follow-up time of 30 days in our study. As expected, cranial procedures resulted in the most new neurological deficits (12.6%), whereas spinal procedures had a rate of 1.6%. This is in line with data published in the literature (7.4% and 2.9%, respectively [16]; 13% [10]). It would indeed be interesting to have follow-up data of these patients, as it is expected that the symptoms should improve or may resolve completely over time.

Taken together, 18.5% of patients treated in our institution had one or more AEs including 6% of patients who had to undergo revision surgery within 30 days after initial surgery. To put these number into perspective, we conducted a literature research compiling all studies of the last 15 years, which reported on adverse events or post-operative complications in neurosurgery on an institutional or multi-center level and represented a “general” neurosurgical patient population (Table 4). Studies with redundant study populations (e.g., 5- vs. 10-year follow-up) or studies focusing on only one pathology were omitted. Ten studies were retrospective in character, whereas seven were prospectively conducted. Important to say, that although retrospectively analyzed, some studies used a prospectively curated database to assess AEs. One third of studies used regularly held morbidity and mortality conferences (MMC) to record and assess AEs. Four studies used administrative data such as readmission rate, reoperation rate, mortality, and length of stay to identify AEs. Rotman et al. discussed the validity of such data and found that there was a discrepancy between such hospital quality metric standards and AEs identified in the morbidity and mortality conference especially in the quality of adverse events [21]. More importantly, there was a wide variety of how AEs were classified. While some institutions implemented standardized classification systems such as the therapy associated Clavien-Dindo grade, other authors included the judgment of fellow neurosurgeons and included categories such as preventable, avoidable, and expectable. Although useful for educational purposes, we believe that such assessment parameters are to be avoided since these notions give room for interpretation and make comparability between neurosurgical centers somewhat difficult [10, 13, 27, 26]. Hence, in this study, the category “new neurological deficit” was used to circumvent this problem. When averaging the rate of AEs across all studies excluding those only considering readmissions or reoperations, a rate of 22.5% of patients suffering from an AE was yielded. Acknowledging the heterogeneity in methodology, our results showed an AE rate of 18.5%, which is below average.

**Table 4:** Literature review of studies of the last 15 years on adverse events in neurosurgery on an institutional or multi-center level representing a “general” neurosurgical patient population

Author	Year	Study design	*n* =	Data acquisition	AE classification	Rate of AE
Al Saiegh	2020	Retrospective (2 years)	7418	MMC	Mortality, unintended and undesirable diagnostic or therapeutic AE, AE that prolongs hospital stay, outcome with permanent or transient neurologic deficit	14.8%
Boström	2010	Prospective, non-consecutive (1 year)	756	Questionnaire directly post-op (voluntary)	Intra-operative AEs: deviation from optimal course; severity, preventability, and consequence	25%
Buchanan	2014	2-center institutional, retrospective (3 years)	5569	30-day readmission	Surgical and medical complications, problem associated with original diagnosis, neurological decompensation, other, and pain	Readmission 6.9%
Gozal	2019	Prospective (1 year)	2965	MMC	(1) Indication errors, (2) procedural errors, (3) technical errors, (4) judgment errors, and (5) critical events	Reoperation 4%
Houkin	2009	Retrospective (2 years)	643	MMC	Predictability, avoidability, and human error	28.3%
Kashiwazaki	2022	Retrospective (9 years)	3262	MMC	Preventability, personal vs. systems error	10%
Landriel Ibanez	2011	Retrospective (1 year)	1190	MMC	Clavien Dindo grade analogue	14%
Lohmann	2021	Prospective (2 years), brain and spinal tumors	1000	Registry with administrative and clinical data	Nosocomial infections, reoperations, readmissions, and mortality	14.5%
Meyer	2022	Prospective (1 year)	4176	Prospectively recorded database	SAVES-v2, human performance deficiency	25%
Rock	2018	Retrospective, multi-center, (10 years)	175,313	Database	Current procedural terminology codes (CTP)	13.5%
Rotman	2018	Retrospective (3 years)	978	Hospital coding/billing records vs. MMC	Billing records vs. MMC cases	77%
Sarnthein	2022	Prospective (7 years)	8226	Case report form at discharge	Clavien Dindo grade	20%
Schipmann	2019	Retrospective (4 years), brain and spinal tumors	2623	Administrative data	Unplanned readmission rate, reoperation rate, mortality, nosocomial infection rate, and LOS	23.7%
Shah	2013	Retrospective (1 year)	3552	30-day readmission	AE even though best practices were followed, due to progression of their underlying disease, preventable causes	Readmission 6.1%
Steiger	2010	Prospective (5 years)	9885	MMC	Positive with resolution of preoperative symptoms, expected, complicated, dead	7.1%
Stone	2007	Single surgeon, prospective (6-years)	1108	Prospectively recorded database	Type, severity, preventability, and consequence	16.8%
Terrapon	2021	2-center retrospective (7 years)	6071	Prospectively recorded database	Therapy-Disability-Neurology grade	25%

*SAVES-V2*, Spinal Adverse Events Severity System version 2; *MMC*, morbidity and mortality conference; *LOS*, length of stay

### Limitations

We do acknowledge limitations of this study. First, the 30-day follow-up time is an arbitrary cut-off, which has been used throughout the literature. The goal of our database is to identify early adverse events, which can be directly linked to the initial procedure. Mid- and long-term complications such as adjacent level disease after spinal fusion surgery or delayed hydrocephalus after tumor surgery was not subject of this study. Second, underreporting may still be an issue even if our reporting algorithm encompasses several control mechanisms including a peer-review process. Lastly, a more standardized grading system for AEs, such as the therapy-based Clavien-Dindo classification or the newly proposed Therapy-Disability-Neurology grading system, would allow better comparability across neurosurgical centers and has indeed been implemented since the beginning of 2022 in our institution [3, 14, 28].

## Conclusion

Adverse events in neurosurgery are not infrequent and occurred in one in five patients in this study cohort. Therefore, a prospective and continued identification and interpretation of AEs represent the basis for effective quality control in modern day hospital management. For this study, AE data of 2837 patients were prospectively collected, categorized, and analyzed. The results presented in this study comprehensively describe AEs of a high-volume neurosurgical center.

## Data Availability

The data that support the findings of this study are available from the corresponding author upon reasonable request.

## Abbreviations

AE: Adverse event
CSF: Cerebrospinal fluid
ICU: Intensive care unit
IMC: Intermediate care unit
NND: New neurological deficit
OR: Operating room
POPAE: Post-operative adverse event
QM: Quality management
